# Extracellular vesicles secreted by hypoxia pre-challenged mesenchymal stem cells promote non-small cell lung cancer cell growth and mobility as well as macrophage M2 polarization via miR-21-5p delivery

**DOI:** 10.1186/s13046-019-1027-0

**Published:** 2019-02-08

**Authors:** Weihua Ren, Jianfeng Hou, Chenguang Yang, Hanjun Wang, Shuangting Wu, Yabin Wu, Xingpeng Zhao, Chao Lu

**Affiliations:** 1 Medical Examination Center, Luoyang Central Hospital, Zhengzhou University, No.288, Middle Zhongzhou Rd, Xigong District, Luoyang, 471009 China; 2 Department of Oncology, Zhengzhou Central Hospital, Zhengzhou, Henan China; 3 Department of Oncology, Kaifeng Central Hospital, Kaifeng, 475000 China; 4 Imaging Department of Luoyang Orthopedic-traumatological Hospital, Luoyang, 471000 China; 5 Department of Anesthesiology, Luoyang Central Hospital, Zhengzhou University, Luoyang, 471009 China

**Keywords:** Mesenchymal stem cells, Extracellular vesicles, Hypoxia, miR-21-5p, Lung cancer, Macrophage polarization

## Abstract

**Objective:**

To investigate the lung cancer-promoting mechanism of mesenchymal stem cell-secreted extracellular vesicles (MSC-EV).

**Methods:**

EV were isolated from culture media of human bone marrow-derived MSCs that were pre-challenged with or without hypoxia (referred to as H-EV and N-EV, respectively). After treatment with N-EV or H-EV, A549 and H23 cell proliferation, apoptosis, trans-well invasion and epithelial-to-mesenchymal transition (EMT) were examined. Polarization of human primary monocytes-derived macrophages with or without N-EV or H-EV induction were analyzed by flow cytometry and ELISA. PTEN, PDCD4 or RECK gene was overexpressed in A549 cells, while miR-21-5p was knocked down in MSCs, A549 or H23 lung cancer cells or primary monocytes by miR-21-5p inhibitor transfection. Protein level of PTEN, PDCD4, RECK, AKT or STAT3 as well as phosphorylation level of AKT or STAT3 protein were assayed by western blot. Tumorigenicity of A549 and H23 cells with or without MSC-EV co-injection was assayed on immunocompromised mice. The xenograft tumor were examined for cell proliferation, angiogenesis, apoptosis and intra-tumoral M1/M2 macrophage polarization.

**Results:**

Comparing to N-EV, H-EV treatment significantly increased A549 and H23 cell proliferation, survival, invasiveness and EMT as well as macrophage M2 polarization. MiR-21-5p knocked down significantly abrogated the cancer-promoting and macrophage M2 polarizing effects of H-EV treatment. H-EV treatment downregulated PTEN, PDCD4 and RECK gene expression largely through miR-21-5p. Overexpressing PTEN, PDCD4 and RECK in A549 cells significantly reduced the miR-21-5p-mediated anti-apoptotic and pro-metastatic effect of H-EV, while overexpressing PTEN in monocytes significantly reduced macrophage M2 polarization after induction with the presence of H-EV. H-EV co-injection significantly increased tumor growth, cancer cell proliferation, intra-tumoral angiogenesis and M2 polarization of macrophages in vivo partially through miR-21-5p.

**Conclusions:**

Increased miR-21-5p delivery by MSC-EV after hypoxia pre-challenge can promote lung cancer development by reducing apoptosis and promoting macrophage M2 polarization.

**Electronic supplementary material:**

The online version of this article (10.1186/s13046-019-1027-0) contains supplementary material, which is available to authorized users.

## Introduction

Mesenchymal stem cells (MSCs, also known as mesenchymal stromal cells) are progenitor cells known for their inflammation resolving, wound healing and homeostasis maintaining properties with high heterogeneity [1]. The therapeutic value of MSCs against various inflammatory diseases and disorders is being tested in clinical trials and preclinical studies, and multiple researches have demonstrated that these anti-inflammatory and immunoregulatory effects of MSCs is generally mediated in a non-contact fashion, mostly via extracellular vesicle secretion (EV) [2, 3]. EV is composed of secreted small particles including exosome and microvesicles with diameter less than 1000 nm, mediating intercellular communications by transferring proteins and nucleotides from donor cells to recipient cells, thus regulating gene expression and intracellular signal transduction in the latter [4, 5]. Previous researches have demonstrated that EV secreted by MSCs (MSC-EV) mediate the anti-inflammatory and cytoprotective effect of MSCs against ischemia-reperfusion injury or other acute injuries in different tissues by inhibiting apoptosis, immune cell-induced cell death and pro-inflammatory response via microRNA delivery [6]. A recent research by Luther et al. showed that MSC-EV protect cardiomyocytes from apoptosis during myocardial infarction via miR-21-5p transfer, which inhibits the protein expressions of several pro-apoptotic genes such as PTEN, PDCD4 and Fas ligand, while Song et al. demonstrated that IL-1β-treated MSCs secrete exosomes with increased miR-146a package, which is transferred into macrophages and promote their M2 polarization [7, 8].

As a distinct type of inflammatory disease, tumors are often considered as “wounds that never heal” on the immunological prospective. In general, MSCs, as they do to the site of other tissue wound, are found to migrate to the tumor microenvironment, where they transformed into tumor associated MSCs and potently inhibit anti-tumor immune attack via EV secretion [9, 10]. However, researchers might draw different conclusions on the participation of MSCs in oncogenesis and development of specific type of cancers. For example, a recent study by Pan et al. suggested that treatment of human bone marrow derived MSCs (hBM-MSC) secretome could inhibit proliferation and metastatic capacity of A549 non-small cell lung cancer cells, and Kalimuthu et al. reported that EV secreted by mouse BM-MSC could inhibit lewis lung cancer development in vivo, while the lung cancer-promoting role of MSCs or EV secreted by MSCs is also demonstrated [11–14]. We believe that these controversies might result from the high heterogeneity of MSCs of different tissue origin, different experimental settings and the complexity of the tumor microenvironment (TME), where a variety types of non-cancer cells were found, such as cancer-associated fibroblasts, tumor infiltrating lymphocytes and, most abundantly, the “M2” like tumor associated macrophages, which is currently the best appreciated immunosuppressive and cancer promoting immune cell in the TME.

The present research was inspired by a recent publication by Lo Sicco et al., who reported that MSC-EV treatment could polarize macrophage towards the M2 phenotype, and this effect could be enhanced when MSCs were challenged with hypoxic culture condition (1% O_2_ atmosphere) before EV isolation [15]. TME is often composed of hypoxic area due to the distorted angiogenesis, and hypoxia plays pivotal role in the establishment of the immunosuppression in the TME [16]. Several other researchers also reported the enhanced cytoprotective and anti-inflammatory effect of EV secreted by MSCs after hypoxia pre-challenge in different tissue damage models [17–19]. Particularly, Cui et al. compared the expression level of several miRNA known as hypoxia inducible in EV secreted from hypoxia pre-challenged and un-challenged MSCs, showing that miR-21-5p is the most significantly upregulated miRNA in EV after hypoxia challenge [18]. MiR-21-5p has been demonstrated as a powerful anti-apoptotic and cancer-promoting miRNA, indicated as prognostic marker in lung cancer and other cancer types [20–22]. Canfrán et al. and Xi et al. both reported that miR-21-5p depletion favors the macrophage M1 polarization, enhancing the macrophage-mediated pro-inflammatory and tumoricidal response [23, 24].

Based on these previous findings we speculated that MSCs might be reshaped by hypoxia, secreting EV that contain increased level of hypoxia inducible miR-21-5p, which can be transferred to recipient cells such as cancer cells and macrophages, thus promoting cancer cell survival and immunosuppression in the TME. In the present research we compared the influence of treatment with EV secreted by MSCs cultured in normal conditions or challenged with hypoxia on A549 and H23 cancer cell growth, metastatic activity as well as macrophage polarization. Our data indicated that encountering with hypoxic environment confers the cancer promoting effect on MSC-EV; EV secreted by MSCs after hypoxia pre-challenge could significantly promote cancer cell survival and metastasis in vitro, tumor development in vivo and macrophage M2 polarization via miR-21-5p delivery.

## Materials and methods

### Cell culture and preparation

A549 and H23 human NSCLC cells as well as human bone-marrow derived MSCs (hBM-MSCs) were purchased from American type culture collection (Manassas, USA). hBM-MSCs were cultured using a human MSCs proliferation kit (STEMCELL Technologies, Vancouver, Canada) following manufacturer’s instructions. A549 or H23 cells were cultured in DMEM with 1000 mg/L D-Glucose (STEMCELL Technologies) supplemented with 10% FBS (Thermo Fisher Scientific, Waltham, USA) and 100 U/ml of penicillin/streptomycin (Thermo Fisher Scientific) in a humidified incubator. MiR-21-5p inhibition in A549, H23, hBM-MSCs or isolated monocytes were performed by stable transfection of miR-21-5p inhibitor (GeneCopoeia, Rockville, USA). A non-targeting miRNA inhibitor control (i-NC) was also transfected as negative control for miR-21-5p inhibition.

### Isolation of EV from hBM-MSCs culture media

Before EV isolation, hBM-MSCs at about 85% confluent were thoroughly washed with complete culture medium and then cultured under 20% O_2_ (normoxic) OR 1% O_2_ (hypoxic) conditions for 24 h, followed by EV isolation as described by Lo Sicco et al. [15] with minor modifications. Briefly the cell culture media was centrifuged at 300×g for 5 min at room temperature, 2000×g for 30 min at 4 °C and 10,000×g for 30 min at 4 °C to remove cells and cell debris. The supernatant was centrifuged at 100,000×g for 60 min at 4 °C, and the pellet was resuspended in sterile PBS, followed by another centrifugation at 100,000×g for 60 min at 4 °C to isolate MSC-EV. MSC-EV were re-suspended in sterile PBS before use. MSC-EV aliquots were equilibrated for EV density based on their total protein concentration measured using a BCA protein assay kit (GeneCopoeia) following manufacturer’s instructions. EV precipitation from cell culture media was confirmed by western blot detecting EV marker CD9 and CD81 (Additional file 1: Figure S1).

### Demonstration of EV uptake by A549 or PBMCs

To demonstrate the uptake of EV by monocytes or A549 cells, we incubated the EV secreted by hypoxia-challenged or naïve MSCs with 40 μM of CFSE dye (Thermo Fisher Scientific) at 30 °C for 1 h, and the EV were pelleted down by centrifugation at 100,000×g for 60 min at 4 °C and washing with PBS. Recipient cells were incubated with re-suspended EV for 24 h under cell culture condition, and cells with or without EV incubation were subject to flow cytometry detecting cellular fluorescence at 492/517 nm (Additional file 2: Figure S2).

### Cell functional assays

Cell proliferation assay was performed using a CCK-8 cell counting kit (Dojindo, Kumamoto, Japan) following manufacturer’s instructions. Briefly, A549 or H23 cells were seeded on 96 well plate in combination with different MSC-EV, and viable cells were measured by incubation with CCK-8 solution for 1 h under cell culture condition followed by measuring the optimal density at 450 nm using a microplate reader. Cell apoptosis was analyzed by flow cytometry detecting FITC-Annexin V/PI positive staining rate on cell surface of A549 or H23 cells after indicated treatment. For hypoxia or cisplatin challenge, A549 or H23 cells were pre-treated with different MSC-EV for 24 h, followed by incubation under 1% O_2_ (hypoxic) conditions or with 5 μM of cisplatin (Tocris Bioscience, Minneapolis, USA) for 24 h, respectively. Cells with Annexin V positive staining were considered apoptotic cells and those with Annexin V^−^PI^+^ being necrotic cells. Trans-well invasion assay was performed using matrigel invasion chambers (Thermo Fisher Scientific). After treatment with different MSC-EV for 24 h in a serum-free environment, A549 or H23 cells were lifted by trypsin (Raybiotech, Norcross, USA), and equal amount of A549 or H23 cells from each treatment group were seeded in the invasion chamber, which was inserted in complete culture medium. Cells were incubated for another 12 h before A549 or H23 cells migrated to the bottom of the chamber being stained with 0.5% methylrosaniline chloride.

### Macrophage induction and analysis

Human monocytes were isolated from peripheral blood donated by healthy individuals. Briefly, peripheral blood mononuclear cells (PBMC) were first isolated by ficoll 400 gradient (Sigma-Aldrich, St. Louis, USA) centrifugation, and monocytes from PBMC were isolated using human monocyte enrichment kit (STEMCELL Technologies) following manufacturer’s instructions. Isolated monocytes were induced with 20 ng/mL of macrophage colony stimulating factor (R&D Systems, Minneapolis, USA) with the presence of different MSC-EV in RPMI-1640 medium supplemented with 10% FBS (STEMCELL Technologies) and 100 U/ml of penicillin/streptomycin (Thermo Fisher Scientific). After 5 days of induction with fresh media and induction environment replaced on day 3, cells were subject to flow cytometry detecting their surface expression level of CD68 and CD163/CD206 or CD40/CD86 using fluorescent antibody purchased from BD Biosciences (San Jose, USA). After induction, macrophages were cultured in fresh medium for 2 days, and protein expression level of IL-10, TGF-β, CCL18 and VEGF-α in macrophage culture medium were determined by using a customized ELISA assay kit (MultiSciences, Hangzhou, China) following manufacturer’s instructions.

### RT-qPCR and western blot

MiR-21-5p expression in cells or isolated EV as well as pre-miR-21 expression in cells were analyzed by RT-qPCR using U6 spliceosomal RNA as internal reference. Briefly, total RNAs extracted by TRIsol were reversely transcribed into cDNA using TaqMan Small RNA Assays kits with pre-miR-21-, hsa-miR-21-5p- or RUN6B-specific RT primers (Invitrogen, Waltham, USA). Primers for pre-miR-21 are as follows: forward, 5’-TACCTCGAGTGTCTGCTTGTTTTGCCT-3′; reverse, 5’-TACGAATTCTGTTTAAATGAGAACATT-3′. Primers for miR-21-5p are as follows: Forward: 5’-TAGCTTATCAGACTGATGTTGA-3′; reverse: 5’-TGCGTGTCGTGGAGT-3′. Primers for U6 are as follows: forward: 5’-GCTTCGGCAGCACATATACTAAAAT-3′; Reverse: 5’-CGCTTCACGAATTTGCGTGTCAT-3′. Semi-quantification of miR-21-5p or pre-miR-21 expression level was performed using 2^-ΔΔCt^ method. Protein level of N-cadherin, E-cadherin, and Vimentin (NBP1–48309, NBP2–19051 and NBP1–31327, respectively, Novus Biologicals), CD9 and CD81 (NBP2–22187 and NB100–65805, Novus Biologicals) Arginase-1 and iNOS (P05089 and MAB9502, R&D Systems), PTEN (4C11A11, BioLegend, San Diego, USA), PDCD4 (NBP2–26138, Novus Biologicals, Littleton, USA), RECK (MA5–14781, Invitrogen), AKT (ab8805, Abcam, Cambridge, USA), STAT3 (NBP2–22471, Novus Biologicals), GAPDH (NB300–221, Novus Biologicals) as well as Ser473 phosphorylation of Akt protein (649,001, BioLegend) and Tyr705 phosphorylation of STAT3 (AF4607, R&D Systems) in cell lysate was analyzed by western blot. Protein expression level of each gene was normalized to that of GAPDH by gray scale analysis using ImageJ software (Ver. 1.52b).

### In vivo assay using xenograft model

Animal use and experimental methods were approved by the Ethics review committee of Luoyang Central Hospital Affiliated to Zhengzhou University. Xenograft model was established by injecting A549 or H23 cells subcutaneously on the back of NU/J nude mice (Jackson laboratory, Bar Harbor, USA). Briefly, 48 male mice at age of 4–6 weeks with roughly equal body weight were randomly assigned to 8 groups. MSC-EV from differentially treated MSCs were re-suspended in sterile PBS and aliquoted with the same total protein concentration (as described above) and were intravenously injected in each experimental mouse every 2 days after A549 or H23 cell injection, and tumor volume on each animal was monitored using a Vernier caliper. Mice were euthanized at day 30 after A549 or H23 cell transplantation, and tumors were dissected and weighted. Cell proliferation and intra-tumoral angiogenesis was determined by immunohistochemical staining of Ki67 (ab15580, Abcam) and CD31 (ab28364, Abcam), respectively, in xenograft tumor derived tissue section. Cell apoptosis in tumor tissue was detected using a terminal deoxynucleotidyl transferase dUTP nick end labeling (TUNEL) Andy Fluor™ 488 apoptosis detection kit (Genecopoeia) following manufacturer’s instructions with minor modifications. Briefly, dissected tumor tissue buffered in pre-chilled PBS was grinded through a cell strainer to make single cell suspension; after centrifugation, cell pellet was resuspended in fixation buffer, and same number of cells were loaded on 96-well plate for fluorescent TUNEL staining and assessment with a microplate reader. Murine macrophages within the xenograft was isolated from the single cell suspension using anti-mouse F4/80 microbeads (Miltenyi Biotec, Auburn, USA) following the product manual. Evaluation of M1/M2 state of these macrophages was performed by detecting Arginase-1 and iNOS protein level by western blot.

### Statistical analysis

Statistical analysis was performed using GraphPad Prism software (Ver. 7.04). Student’s t-test was used to evaluate the differences between two groups. Dunnett’s multiple comparisons test was used for comparing different experimental group to one control group, and Tukey’s multiple comparisons test was used for multigroup comparison. *P* < 0.05 was considered statistically significant. For in vitro experiments, each group of data represents 3 independent replicates and were presented as mean ± standard deviation (SD) unless otherwise indicated. For in vivo experiments, each group of data represents 6 independent replicates.

## Results

### Extracellular vesicles secreted by hypoxia pre-challenged mesenchymal stem cells promoted cell proliferation, survival, mobility and EMT of NSCLC cells as well as macrophage M2 polarization in vitro

**Fig. 1:**
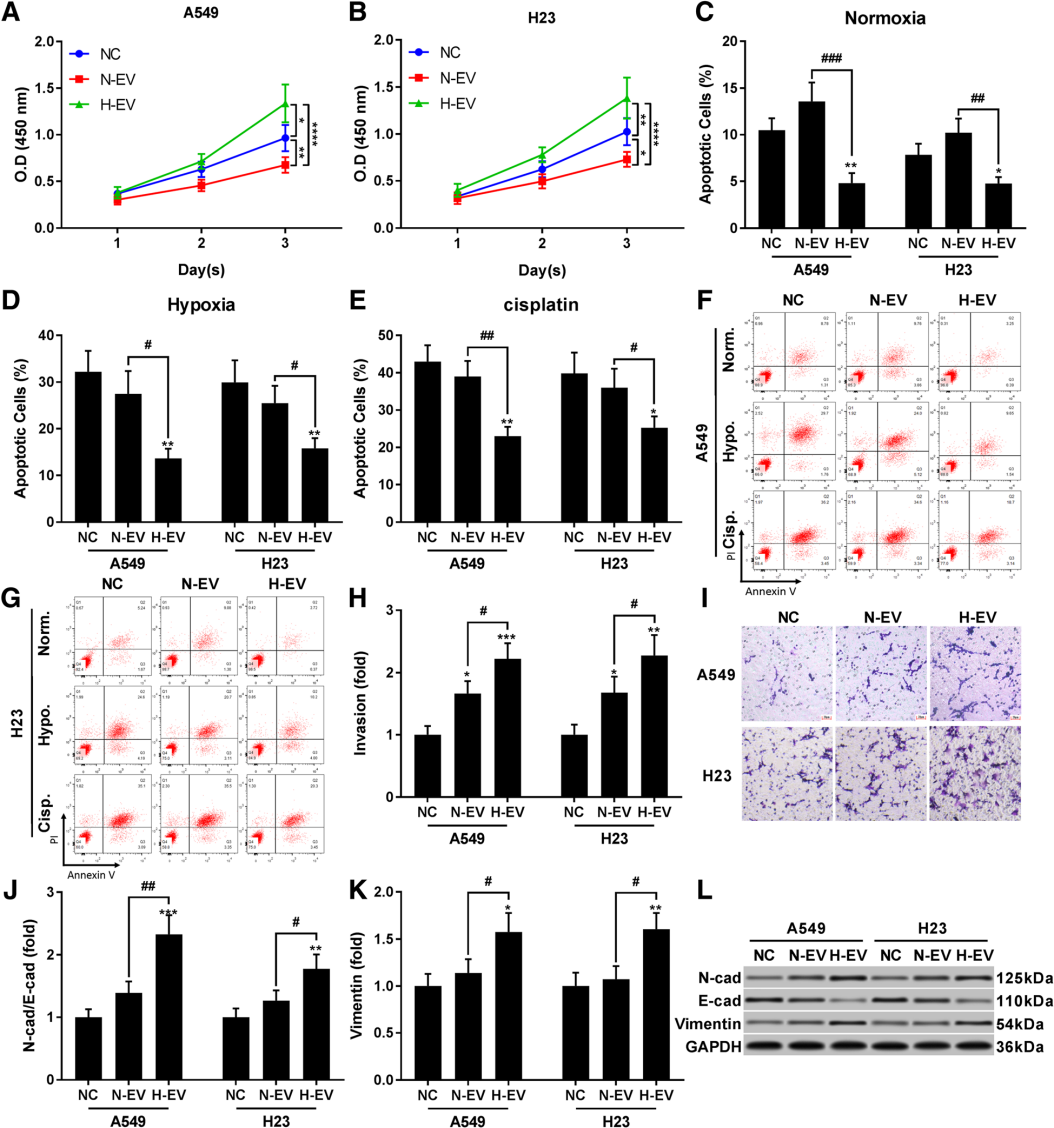
Extracellular vesicles (EV) secreted by hypoxia pre-challenged mesenchymal stem cells (MSCs) promote A549 and H23 cell proliferation, survival, mobility and EMT in vitro. Cells were pre-treated with EVs secreted by naïve (N-EV) or hypoxia pre-challenged (H-EV) MSCs. **a** and **b**, CCK-8 cell proliferations assay evaluating viable cell count at different timepoints. **c**–**g**, A549 and H23 cell apoptosis under normal, hypoxia challenge (1% O_2_) or cisplatin challenge (5 μM) after N-EV or H-EV treatment. Hypoxia or cisplatin challenge was performed for 24 h before analysis. **h**–**i**, trans-well assay evaluating A549 and H23 cells invasion after N-EV or H-EV treatment. A549 or H23 cells treated with PBS vehicle were used as negative control (NC). Bar in I indicates 20 μm. **j**–**l**, western blot detecting epithelial and mesenchymal marker in A549 and H23 cells after treatment with N-EV or H-EV. Statistical analysis results by Student’s *t* test was marked by # and that by Dunnett’s test were marked by *. * or #, *p* < 0.05; ** or ##, *p* < 0.01; ***, *p* < 0.001

Influences of mesenchymal stem cells on lung cancer development or the underlying mechanism of action remains unsettled. In the present research, we first determined the influence of treatment with isolated EV secreted by MSCs cultured under normoxic condition (N-EV) or challenged by hypoxia for 1 h (H-EV) on cell proliferation, survival, migration and invasion capacity of A549 and H23 NSCLC cells in vitro (Fig. 1). CCK-8 cell counting assay suggested that, while treatment with N-EV decreased proliferation of A549 and H23 cells, treatment with H-EV significantly increased A549 and H23 cell proliferation in vitro. Similarly, treatment with N-EV showed a trend in increasing apoptosis of A549 and H23 cells, while H-EV treatment significantly reduced A549 and H23 cell apoptosis in in vitro culture. We also tested the influence of treatment with N-EV or H-EV on apoptosis of A549 and H23 cells cultured with hypoxia or cisplatin challenge. N-EV treatment showed no significant influence on apoptosis of A549 or H23 cell challenged by hypoxia or cisplatin, both of which were significantly reduced by H-EV treatment. Trans-well assay results suggested that treatment with EV secreted by MSCs could significantly increase migration and invasion capacity of A549 and H23 cells in vitro, and these effects were further enhanced when MSCs were pre-challenged by hypoxia. Western blot results suggested that treatment with EV secreted by MSCs that were challenged with hypoxia but not those from naïve MSCs significantly increased EMT of A549 and H23 cells, marked by the increase in mesenchymal phenotype marker protein N-cadherin and Vimentin. These data suggested that H-EV treatment could promote A549 and H23 cell survival in various conditions in vitro, and cell mobility and EMT-promoting effect of MSC-EV on A549 and H23 cells can be enhanced by hypoxia pre-challenge on MSCs.

**Fig. 2:**
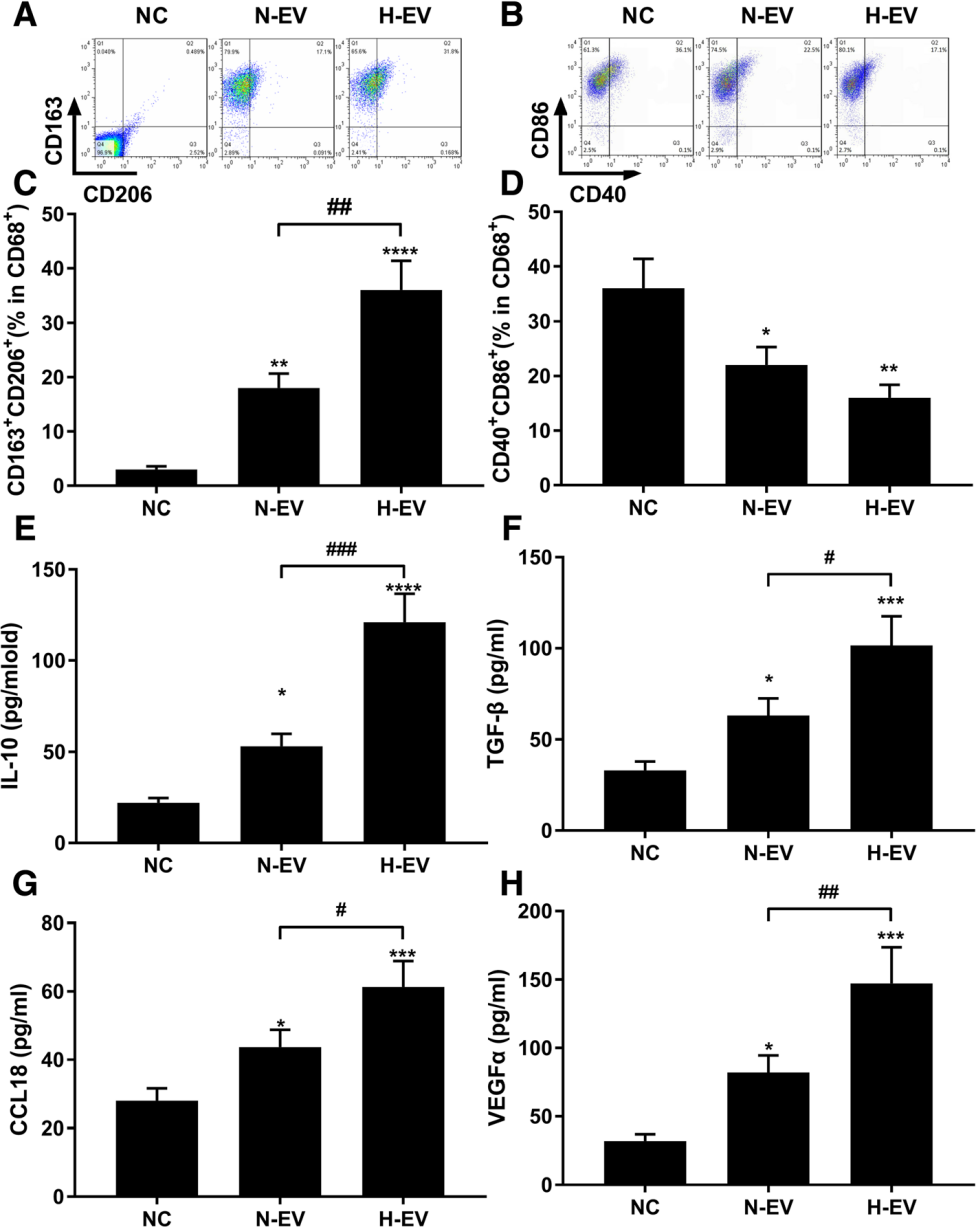
Extracellular vesicles (EV) secreted by normal-cultured (N-EV) or hypoxia pre-challenged mesenchymal stem cells (MSCs) promote macrophage M2 polarization in vitro. Human monocytes freshly isolated from peripheral blood were induced with 20 ng/ml of M-CSF for 5 days with or without the presence of MSC-EV. **a**–**d**, flow cytometry determining the percentage of CD163^+^CD206^+^ cells or CD40^+^CD86^+^ cells among total CD68^+^ cells after induction. **e**–**h**, ELISA detecting protein expression of IL-10, TGF-β, CCL-18 and VEGF-α in macrophage culture medium after induction. Macrophages induced without MSC-EV were used as negative control (NC). Statistical analysis results by Student’s *t* test was marked by # and that by Dunnett’s test were marked by *. * or #, *p* < 0.05; **, *p* < 0.01; ***, *p* < 0.001

The inflammation-resolving and tumor promoting effect of MSCs has been suggested to be dependent on modulating macrophage polarization. We therefore investigated the influence of N-EV or H-EV on macrophage polarization by treating peripheral blood monocytes with either N-EV or H-EV during induction of their differentiation into macrophage using M-CSF (Fig. 2). Flow cytometry results suggested that macrophages induced with M-CSF in combination with N-EV showed significant higher expression of M2 macrophage-related cell surface marker, namely CD163 and CD206, and macrophages induced with M-CSF in combination with H-EV further increased their CD163 and CD206 surface expression, while the M1 macrophage-related marker CD86 and CD40 were decreased by N-EV or H-EV co-induction. Enzyme-linked immuno-sorbent assay further confirmed the significant upregulation of M2 macrophage related secretory proteins, including IL-10, TGF-β, CCL18 and VEGF-α, in culture medium of macrophages induced by N-EV, which were further increased by H-EV induction. These data clearly suggested that MSC-EV could promote macrophage M2 polarization, which can be further enhanced by hypoxia pre-challenge on MSCs.

### H-EV treatment promote A549 cell survival and mobility as well as macrophage M2 polarization in vitro by miR-21-5p delivery

**Fig. 3:**
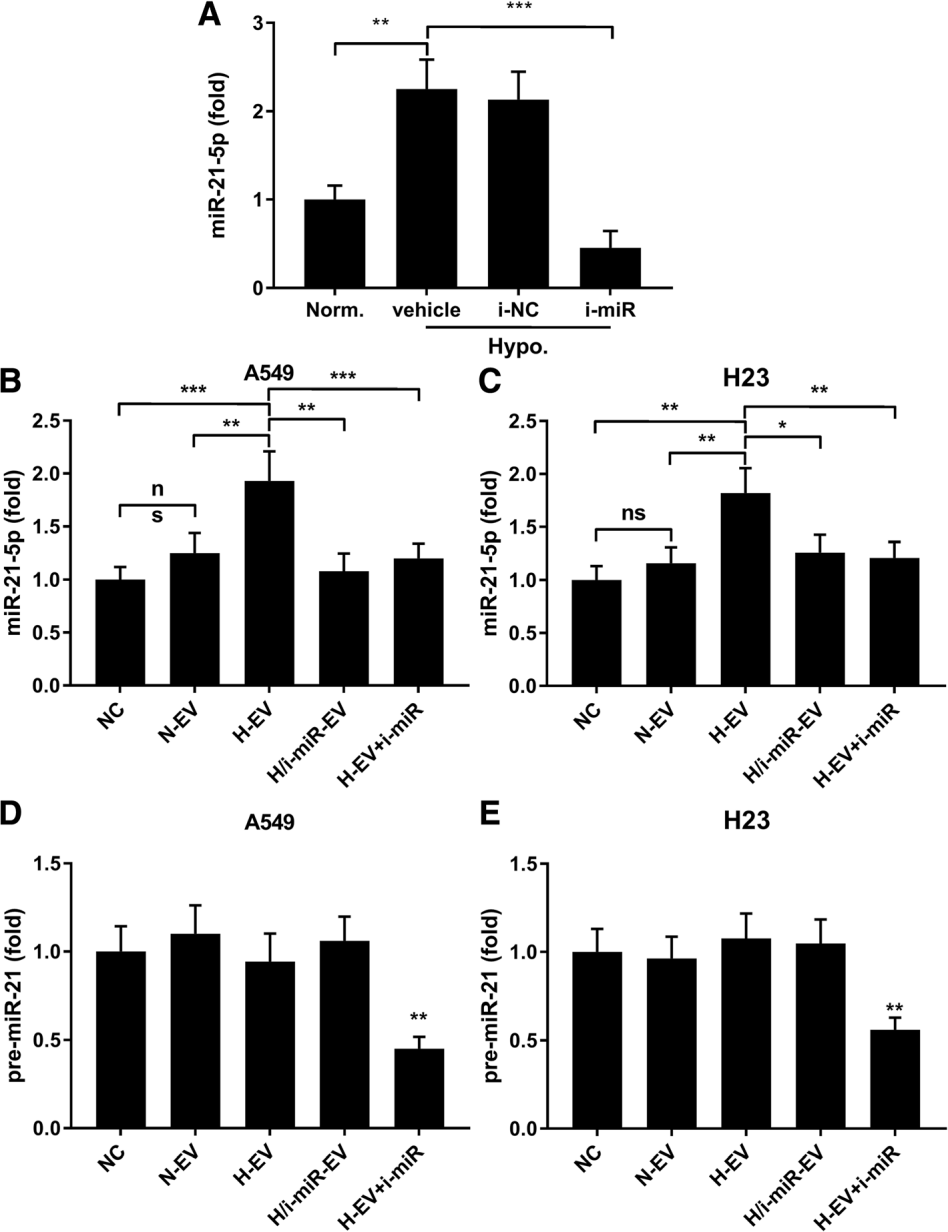
MiR-21-5p was delivered into NSCLC cells in vitro by H-EV. **a**, RT-qPCR detecting miR-21-5p expression level in EV isolated from MSCs after various of treatment. i-miR or i-NC, MSCs were transfected with miR-21-5p inhibitor or microRNA inhibitor negative control before hypoxia challenge and EV isolation. Vehicle, MSCs were treated with transfection reagent without the vector. MSCs cultured in normoxic condition (Norm.) were used as negative control. **b**, **c**, RT-qPCR detecting miR-21-5p expression level in A549 and H23 cells after different treatment. **d**, **e**, RT-qPCR detecting pre-miR-21 expression level in A549 and H23 cells after different treatment. H/i-miR-EV, A549 or H23 cells were treated with EV secreted by miR-21-5p-inhibited, hypoxia pre-challenged MSCs; H-EV + miR, A549 or H23 cells were transfected with miR-21-5p inhibitor before H-EV treatment. Untreated A549 or H23 cells were used as negative control (NC). Tukey’s test was used for statistical analysis. *, *p* < 0.05; **, *p* < 0.01; ***, *p* < 0.001

Precious reports have described the significant upregulation of miR-21-5p in EV secreted by MSCs induced by hypoxia challenge. MiR-21-5p is a well-studied “oncomiR” in multiple types of cancers with PTEN, PDCD4 and RECK as its best-known target genes. PTEN and PDCD4 have been previously shown to impede cancer cell growth and facilitate apoptosis, while RECK could reduce cancer cell mobility by deactivating matrix metalloproteinases. To verify whether miR-21-5p was involved in H-EV promoting cell proliferation, survival and mobility of A549 cells, we constructed miR-21-5p knockdown MSCs, A549 and H23 cells by miR-21-5p inhibitor transfection. Hypoxia challenge significantly increased miR-21-5p expression level in MSC-EV, which was largely obliterated by miR-21-5p inhibition in MSCs (Fig. 3a). Treatment with N-EV showed no significant influence on miR-21-5p expression level in A549 or H23 cells, but treatment with H-EV significantly increased miR-21-5p in these two NSCLC cells, which can be significantly reduced by miR-21-5p inhibition in either MSCs, A549 or H23 cells (Fig. 3b and c). To verify that these miR-21-5p increase in A549 and H23 cells was due to MSC-EV delivery, we examined pre-miR-21 expression level in A549 and H23 cells under various treatment in Fig. 3b and c. Treatment with different MSC-EV showed no significant impact on pre-miR-21 expression level in A549 or H23 cells, but miR-21-5p inhibitor transfection significantly reduced pre-miR-21 expression in A549 and H23 cells despite H-EV treatment (Fig. 3d and e). These data suggested that hypoxia challenge could significantly increase miR-21-5p expression level in MSC-EV, and treatment with H-EV could significantly increase miR-21-5p in NSCLC cells primarily by EV delivery but not by induction of miR-21-5p expression in the recipient cells.

**Fig. 4:**
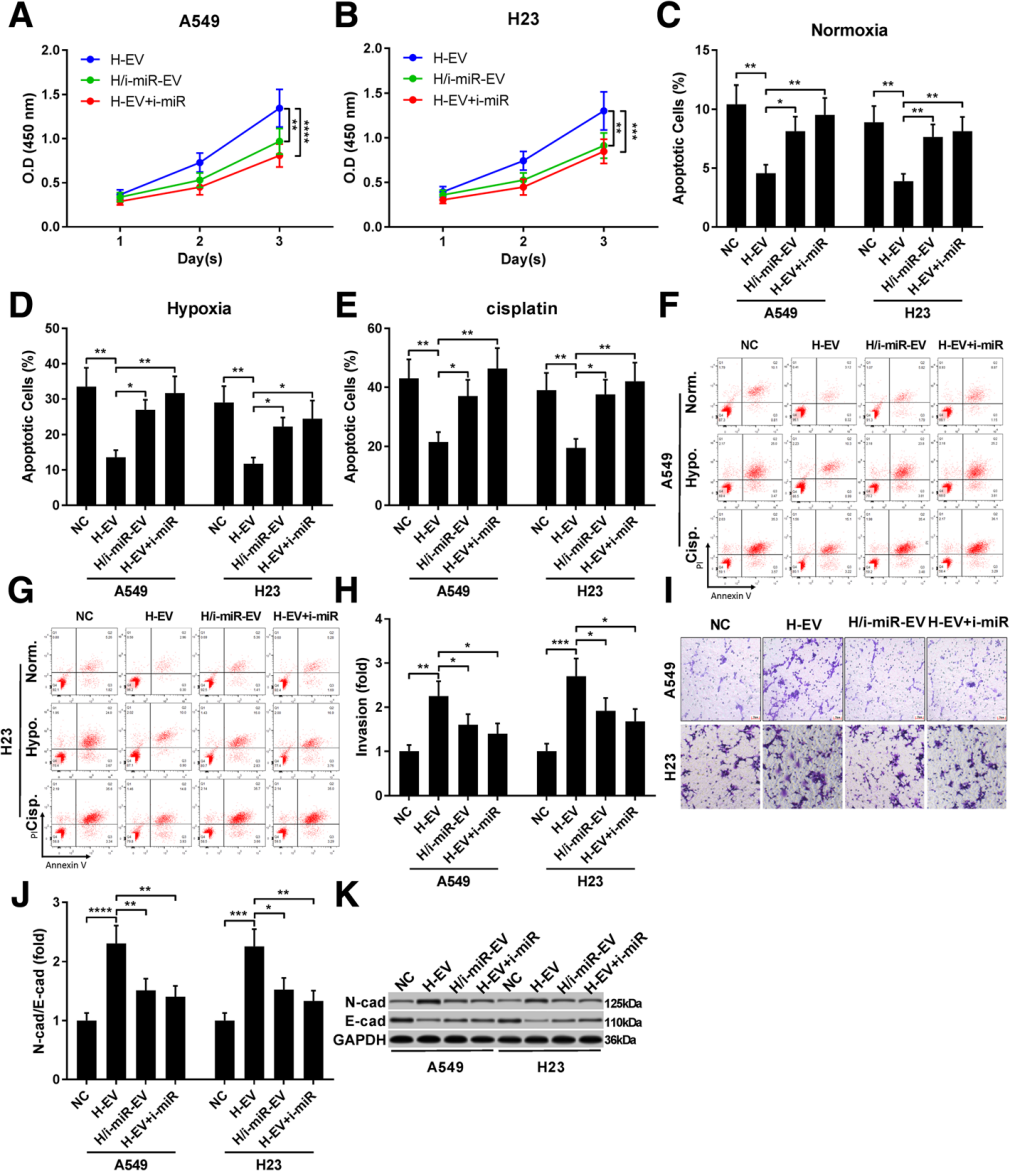
H-EV promoted A549 cell proliferation, survival ad mobility in a miR-12-5p-dependent manner. A549 or H23 cells were transfected with miR-12-5p inhibitor and treated with H-EV. **a**-**k**, cell functional assays were performed as described in Fig. 1. Non-treated A549 or H23 cells were used as negative control (NC). Bar in **i** indicates 20 μm. Data in **h** and **j** were presented as fold change comparing to NC. Tukey's test was used for statistical analysis. *, *p* < 0.05; **, *p* < 0.01; ***, *p* < 0.001

We next investigated whether miR-21-5p inhibition could reduce the effect of H-EV as demonstrated in Fig. 1 (Fig. 4). CCK-8 cell counting assay results suggested that miR-21-5p inhibition in either MSCs, A549 or H23 cells significantly reduced A549 and H23 cell proliferation increased by H-EV treatment, while reduction of A549 and H23 apoptosis under normal, hypoxia or cisplatin challenging condition by H-EV was largely abrogated by miR-21-5p inhibition. Trans-well assay results also suggested that increase in A549 and H23 cell mobility by H-EV treatment could be inhibited by miR-21-5p inhibitor transfection in MSCs, A549 or H23 cells. Western blot results further demonstrated that inhibition of miR-21-5p in either MSCs or NSCLC cells could significantly abrogate the EMT-promoting effect of H-EV on A549 or H23 cells. These data clearly suggested that H-EV treatment promoting cell proliferation, mobility and EMT while inhibiting apoptosis in NSCLC cells was principally mediated by miR-21-5p delivery.

**Fig. 5:**
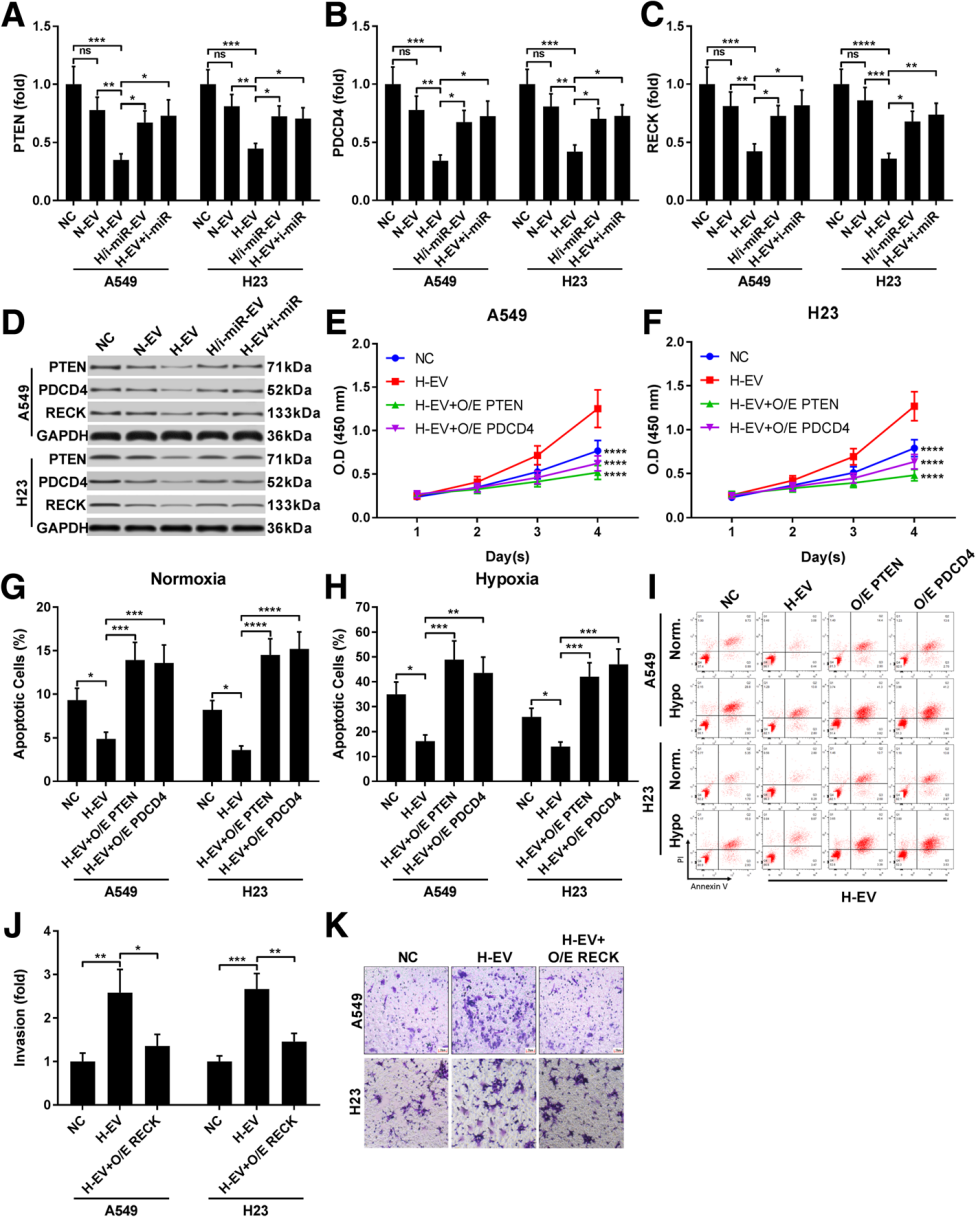
miR-21-5p delivery by H-EV treatment promote NSCLC cell proliferation, survival and mobility by targeting PTEN, PDCD4 and RECK. **a**–**d**, western blot detecting PTEN, PDCD4 and RECK protein expression level in A549 or H23 cells after different treatment as described in Fig. 4. **e**–**k**, cell functional assay as described in Fig. 1. A549 or H23 cells with or without PTEN, PDCD4 or RECK gene overexpression (O/E) were tested. Wild type, non-treated A549 or H23 cells were used as negative control (NC). Bar in K indicates 20 μm. Tukey’s test was used for statistical analysis. *, *p* < 0.05; **, *p* < 0.01; ***, *p* < 0.001; ****, *p* < 0.0001

Considering that PTEN, PDCD4 and RECK has been recognized as direct target genes of miR-21-5p, we verified whether inhibition of these genes’ expression was involved in miR-21-5p-mediated effect of H-EV treatment on NSCLC cells. Western blot results showed that protein expression of these three genes were significantly reduced in A549 and H23 cells by H-EV treatment, which could be rescued by miR-21-5p inhibition (Fig. 5a-d). Cell functional assay further demonstrated that overexpression of either PTEN or PDCD4 could dramatically reverse the cell proliferation-promoting and anti-apoptotic effects of H-EV treatment on A549 and H23 cells (Fig. 5e-i), while RECK overexpression inhibited the increase in cell mobility of A549 and H23 cells treated by H-EV (Fig. 5j-k). These data further confirmed that the effect of H-EV treatment on NSCLC cells is based on miR-21-5p targeting cancer suppressing genes such as PTEN, PDCD4 and RECK.

**Fig. 6:**
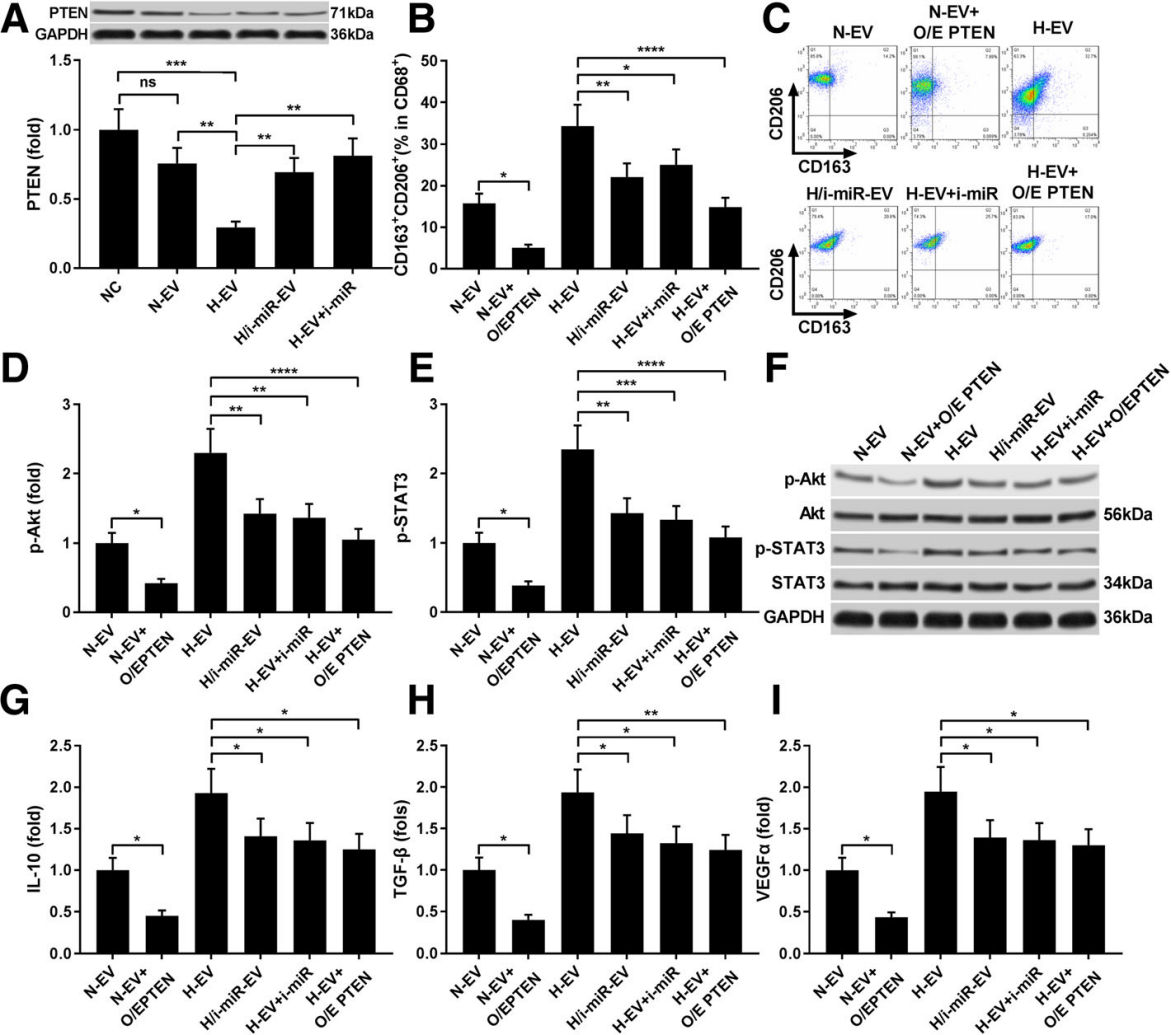
N-EV and H-EV treatment promote macrophage M2 polarization by delivering miR-21-5p that targets PTEN. **a**, western blot analysis of PTEN protein expression level in induced macrophages. H/i-miR-EV, monocytes were induced with the presence of EV secreted by miR-21-5p-inhibited, hypoxia pre-challenged MSCs; H-EV + i-miR, monocytes were transfected with miR-21-5p inhibitor-expressing vector before induction with the presence of H-EV. Macrophages induced without MSC-EV were used as negative control (NC). **b, c**, flow cytometry determining the percentage of CD163^+^CD206^+^ cells among total CD68^+^ cells after induction. N-EV + O/E PTEN or H-EV + O/E PTEN, monocytes were transfected with PTEN overexpressing vector before N-EV or H-EV treatment, respectively. **d**–**f**, western blot detecting Akt and STAT3 protein expression as well as their activating phosphorylation (p-Ser473 for Akt and p-tyr705 for STAT3) in macrophages after induction. **g**–**i**, ELISA evaluating IL-10, TGF-β and VEGF-α in macrophage culture medium after induction. Macrophages induced with the presence of N-EV were used as negative control in **b**–**i**. Tukey’s test was used for statistical analysis. *, *p* < 0.05; **, *p* < 0.01; ***, *p* < 0.001; ****, *p* < 0.0001

Previous reports also demonstrated that PTEN can block macrophage M2 polarization by inhibiting Akt and STAT3 activation. We therefor investigated whether N-EV- and H-EV-induced macrophage M2 polarization is due to miR-21-5p targeting PTEN (Fig. 6). Western blot results first showed that PTEN expression in induced macrophages was significantly reduced by N-EV treatment and further reduced by H-EV treatment, which can be rescued by miR-21-5p inhibitor transfection in either MSCs or monocytes before induction. MiR-21-5p inhibition or PTEN overexpression in monocytes significantly inhibited H-EV in promoting macrophage M2 polarization, as revealed by flow cytometry detecting M2 macrophage surface marker CD163 and CD206 as well as ELISA evaluating M2 macrophage related secretory proteins, including IL-10, TGF-β and VEGF-α. Western blot results further confirmed that macrophage M2 polarization was associated with increased Akt and STAT3 activation, marked by their phosphorylation status, and inhibition of miR-21-5p or PTEN overexpression significantly reduced AKT and STAT3 activation induced by H-EV treatment. These data suggested that N-EV or H-EV treatment induced macrophage M2 polarization by miR-21-5p delivery, which targets PTEN and thus liberates AKT and STAT3 activation.

### H-EV treatment increased tumor cell growth and intratumoral angiogenesis in vivo

**Fig. 7:**
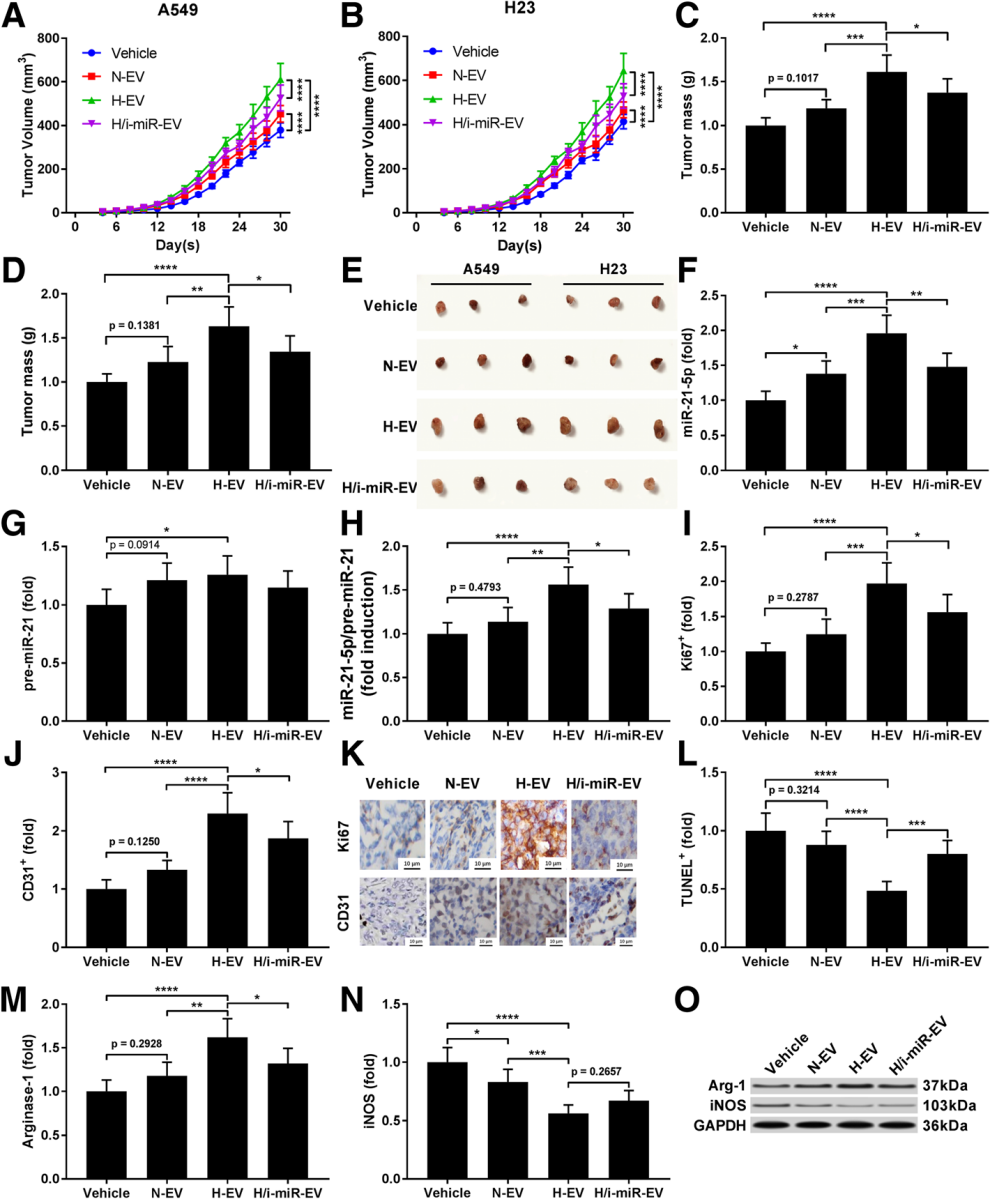
H-EV promoting tumor growth in vivo was reduced by miR-21-5p inhibition. **a** and **b**, tumor volume growth with different MSC-EV treatment in tumor-bearing mice. **c**–**e**, tumor weight at day 30 after A549 or H23 cells transplantation. **f**–**h**, RT-qPCR detecting miR-21-5p and pre-miR-21 expression level in xenograft tumor tissue samples. **i**–**k**, in situ immunohistochemical (IHC) staining of Ki-67 and CD31 in xenograft tumor paraffin section. Bar indicates 10 μm. **l**, terminal deoxynucleotidyl transferase dUTP nick end labeling (TUNEL) in xenograft tumor cells. **m**–**o** western blot detecting murine M1/M2 macrophage marker protein iNOS and Arginase-1 in xenograft tumor. Data in **f**–**o** were obtained using xenograft tumor established using A549 cells. Tukey’s test was used for statistical analysis. *, *p* < 0.05; **, *p* < 0.01; ***, *p* < 0.001; ****, *p* < 0.0001

To investigate the influence of H-EV or N-EV treatment on tumor growth in vivo, xenograft model was constructed by injecting A549 or H23 NSCLC cells subcutaneously in nude mice. Xenograft model mice were intravenously injected with either N-EV, H-EV or H-EV secreted by miR-21-5p inhibited MSCs every two days, and tumor volume was monitored every two days after A549 cell injection using Vernier caliper. Our data demonstrated that injection with N-EV or H-EV significantly accelerated tumor growth, and H-EV injection showed more potent effect comparing to N-EV; notably, the tumor promoting effect of H-EV was impaired by miR-21-5p inhibition in MSCs (Fig. 7a and b). Examining the weight of tumors dissected at day 30 after A549 or H23 cell injection suggested that H-EV injection significantly increased tumor weight, which was impeded by miR-21-5p inhibition in MSCs, while N-EV injection showed no significant effect on tumor weight change (Fig. 7c-e). RT-qPCR detecting miR-21-5p and pre-miR-21 in the xenograft tumors revealed that miR-21-5p in xenograft tumor cells was significantly increased by N-EV treatment and further increased by H-EV treatment, which was largely abrogated by miR-21-5p inhibition in MSCs, but expression of pre-miR-21 was less significantly affected, suggesting the delivery of miR-21-5p into the xenograft tumor cells by MSC-EV (Fig. 7f-h). Immunohistochemical staining examining the dissected tumors showed that H-EV injection significantly increased A549 or H23 cell proliferation and intra-tumoral angiogenesis in the xenograft model, marked by the significant increase in Ki67 and CD31 positive staining rate, respectively, with a significant decrease in TUNEL positive staining rate that suggested a markedly reduction of cell apoptosis; these effects of H-EV injection was significantly reduced by miR-21-5p inhibition in MSCs, while N-EV injection showed no significant influence on cell proliferation, apoptosis or intra-tumoral angiogenesis in the xenograft model (Fig. 7i- l). Results of western blot analyzing arginase-1 and iNOS protein level in murine macrophages isolated from the xenograft tumors suggested that treatment of MSC-EV significantly increased M2 polarization while reducing M1 polarization of intra-tumoral murine macrophages; H-EV showed more potent effects on screwing macrophages towards M2 phenotype in vivo, and this effect was damaged by miR-21-5p inhibition in MSCs (Fig. 7m-o).

## Discussions

EV secreted by MSCs has been suggested as possible anti-cancer drug delivery approach or therapeutic agent by several researches, but different reports on the influence of MSCs or MSC-EV on cancer cell behavior are inconsistent, and the underlying mechanism remains to be discovered. These controversies might be due to the heterogeneity of MSCs of different tissue origins and complexity of the tumor microenvironment. Despite the complicated cross-talk among cancer cells, due to the chaotic angiogenesis in tumor mass, hypoxia in insufficient blood supply area can alter the cell function of cancer cells as well as other cell types residing in the tumor microenvironment. MSCs has been demonstrated to migrate to the tumor microenvironment, where they were reshaped to tumor associated MSCs and exhibit distinct immunosuppressive, angiogenic and cancer promoting functions, which have been suggested to be largely mediated by EV secretion [10]. Previous researches have shown that hypoxia pre-challenge on MSCs increases the promoting effect of MSC-EV on macrophage M2 polarization, and microRNA expression profiles in the MSC-EV can also be significantly altered [15, 18]. Several previously identified hypoxic-inducible miRNAs were found upregulated in MSC-EV after hypoxia pre-challenge, including miR-21-5p, miR-210-3p, miR-23a-3p, etc., and these miRNAs were found to regulate immune cell function and promote cancer development [18]. Caescu et al. found that activation of CSF-1R on murine macrophage facilitated their M2 polarization by inducing miR-21-5p expression, and Xi et al. further demonstrated that miR-21-5p inhibition in macrophages favored their M1 polarization, although the detailed molecular mechanism of miR-21-5p’ action remains to be clarified [25]. Besides, miR-210-3p and miR-23a-3p were also found to reduce CD8^+^ T cell and natural killer cell cytotoxicity towards cancer cells [26–28].

MicroRNA delivery via EV from donor cells to regulate intracellular gene expression and cellular behavior of the recipients has been found under various experimental conditions [29]. The present research started with our hypothesis that MSCs pre-challenged by hypoxia might secrete cancer promoting and immunosuppressive EV, resulting in accelerated tumor development; considering that Cui et al. have demonstrated that miR-21-5p was the miRNA most significantly upregulated in MSC-EV after hypoxia pre-challenge, we inferred that the potential cancer promoting and immunosuppressive role of EV secreted by hypoxia pre-challenged MSCs might be at least in part mediated by miR-21-5p delivery. We first found that H-EV, the EV secreted by hypoxia pre-challenged bone marrow-derived MSCs, significantly increased cell proliferation, survival, mobility and EMT of NSCLC cells in vitro. Interestingly, treatment with EV secreted by un-challenged MSCs (N-EV) showed a trend towards inhibiting NSCLC cell proliferation and survival under normal culture conditions, yet moderately increasing cell mobility, which was similar to what Zhao et al. observed in their recent publication [30]. We next investigated the influence of N-EV or H-EV treatment on macrophage polarization, considering that macrophages are often found the most abundant non-cancerous cell types in the tumor microenvironment, who primarily exhibit “M2” like phenotype and immunosuppressive, anti-inflammatory as well as pro-angiogenic function. Our data showed that treatment with N-EV during macrophage induction increased M2 macrophage marker protein expression and M2 macrophage related protein secretion, and treatment with H-EV showed more potent effect comparing to H-EV. These results are similar to Lo Sicco et al’s recent findings [15].

We further revealed that hypoxia pre-challenge increased miR-21-5p load in MSC-EV, which can be delivered to NSCLC cells. Inhibiting miR-21-5p in either MSCs or NSCLC cells by miR-21-5p inhibitor transfection significantly decreased the promoting effect of H-EV treatment on NSCLC cell proliferation, survival, mobility and EMT, suggesting that these effects of H-EV treatment are largely mediated by miR-21-5p delivery. We further confirmed that the cell proliferation and survival-promoting effect of miR-21-5p on NSCLC cells delivered by H-EV is achieved by PTEN and PDCD4 targeting, and upregulation in NSCLC cell mobility by RECK targeting. Overexpression of these cancer suppressive genes significantly inhibited the effect of H-EV on A549 cells. On macrophage differentiation, our data suggested that increased miR-21-5p delivery by MSC-EV after hypoxia pre-challenge can reduce PTEN expression, thus liberating Akt and STAT3 activation and facilitate macrophage M2 polarization. Using xenograft model, we found that intravenous injection of H-EV significantly increased tumor growth, marked by upregulated cancer cell proliferation and intra-tumoral angiogenesis as well as reduced cell apoptosis. Overall, our data suggested that encountering with hypoxic environment confers the cancer promoting effect on MSC-EV; EV secreted by MSCs after hypoxia pre-challenge could significantly promote tumor development, possibly by increasing cancer cell survival, metastasis and inducing macrophage M2 polarization via miR-21-5p delivery.

Notably, in most of our experiments, miR-21-5p inhibition not completely but only partially reversed the effect of H-EV treatment, especially in our xenograft tumor formation assay, suggesting that other factors loaded in MSC-EV are involved in the cancer promoting and immunoregulatory effect of H-EV as our data suggested. In the present research we also compared the effect of N-EV and H-EV treatment. Besides on macrophage differentiation, N-EV generally showed no statistically significant influence on NSCLC cell behavior or xenograft tumor growth comparing to vehicle treatment. In the present research we found the significant influence of H-EV treatment on macrophage polarization only in in vitro experiments. We also performed IHC staining on M2 macrophage marker protein CD163 and CD206 in tissue sections derived from xenograft tumor and adjacent tissue, but we observed no significant difference in CD163 or CD206 positive staining rate between H-EV treated groups and non-treated counterparts (data not shown). Further verification on the effect of H-EV in promoting macrophage M2 polarization is needed under in vivo conditions.

## Conclusion

Collectively, our data suggested that the cancer promoting effect of EV secreted by hypoxia pre-challenged MSCs was mediated at least partially by miR-21-5p delivery. MiR-21-5p delivered into NSCLC cells by these EV reduced PTEN, PDCD4 and RECK gene expression in NSCLC cells, resulting in increased cell proliferation, survival and mobility. MiR-21-5p delivered into monocytes facilitated their M2 polarization during maturation, resulting in the increase in M2 macrophages in the tumor microenvironment.

## Additional files

Additional file 1: **Figure S1.** MSC-EV precipitation from MSCs culture media was verified by western blot. Input, raw cell culture media. Output, supernatant after EV precipitation. Pellet, EV that were pelleted down by ultra-centrifugation. NC, fresh cell culture media as negative control (processed in parallel). N-EV, EV in naïve MSCs culture media. H-EV, EV in hypoxia pre-challenged MSCs culture media (TIF 2679 kb)

Additional file 2: **Figure S2.** Uptake of MSC-EV by A549 or human circulating monocytes as examples of recipients was confirmed by flow cytometry. Cells were treated with N-EV or H-EV that were labeled with CFSE dye before treatment, and cellular fluorescence was detected by flow cytometry. Cells processed in parallel using vehicle (sterile PBS) was used as negative control. (TIF 4714 kb)

## Abbreviations

EV: Extracellular vesicles
hBM-MSCs: Human bone marrow derived MSCs
MSCs: Mesenchymal stem cells
PBMC: Peripheral blood mononuclear cells
TME: Tumor microenvironment
