# Supplementary figures and images for: Extracellular vesicles secreted by hypoxia pre-challenged mesenchymal stem cells promote non-small cell lung cancer cell growth and mobility as well as macrophage M2 polarization via miR-21-5p delivery
Source: J Exp Clin Cancer Res. 2019 Feb 8;38:62. doi: 10.1186/s13046-019-1027-0 (PMC6367822; doi:10.1186/s13046-019-1027-0)

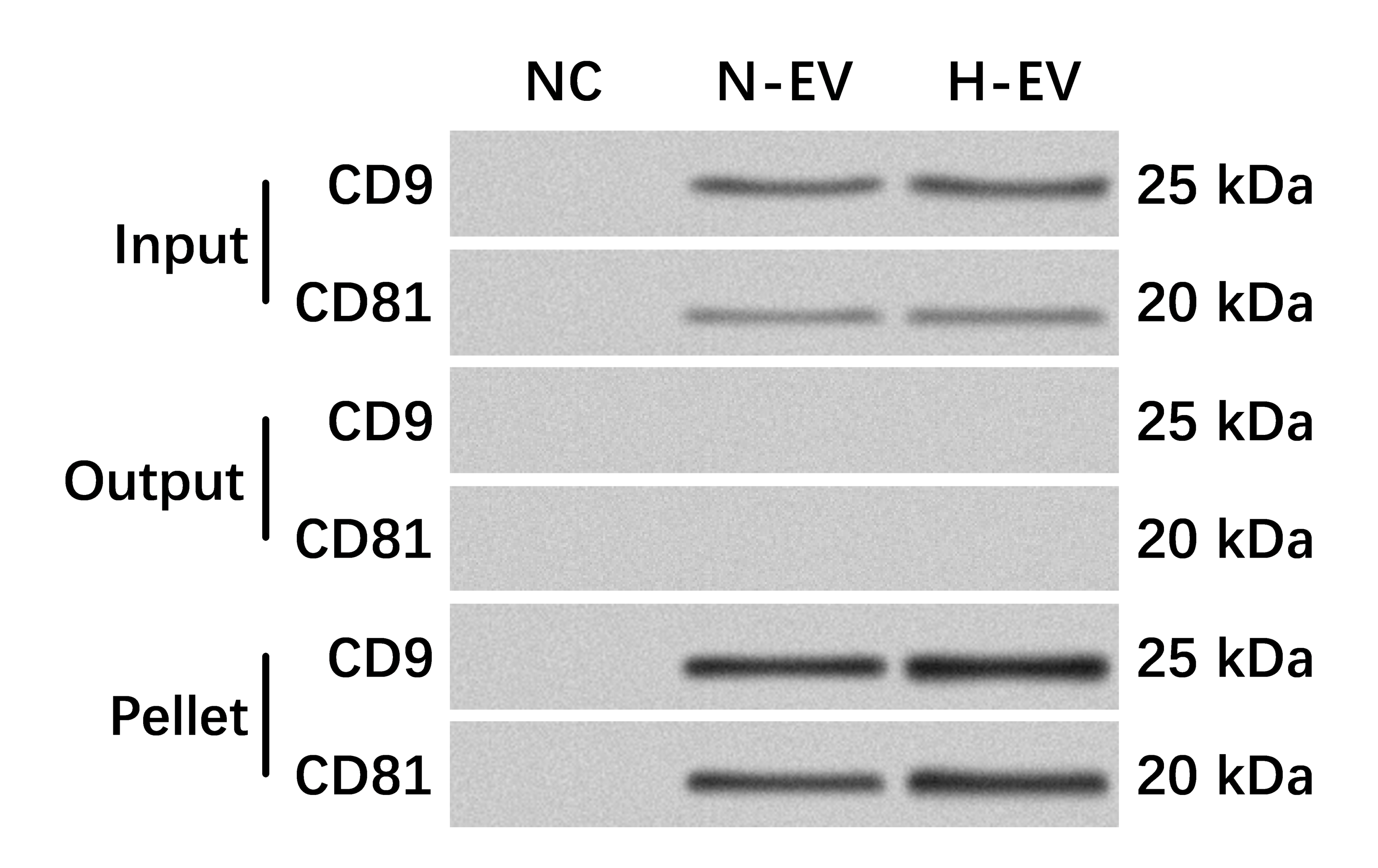

Supplement: Supplementary file 1 — Figure S1. MSC-EV precipitation from MSCs culture media was verified by western blot. Input, raw cell culture media. Output, supernatant after EV precipitation. Pellet, EV that were pelleted down by ultra-centrifugation. NC, fresh cell culture media as negative control (processed in parallel). N-EV, EV in naïve MSCs culture media. H-EV, EV in hypoxia pre-challenged MSCs culture media (TIF 2679 kb) [file 13046_2019_1027_MOESM1_ESM.tif]

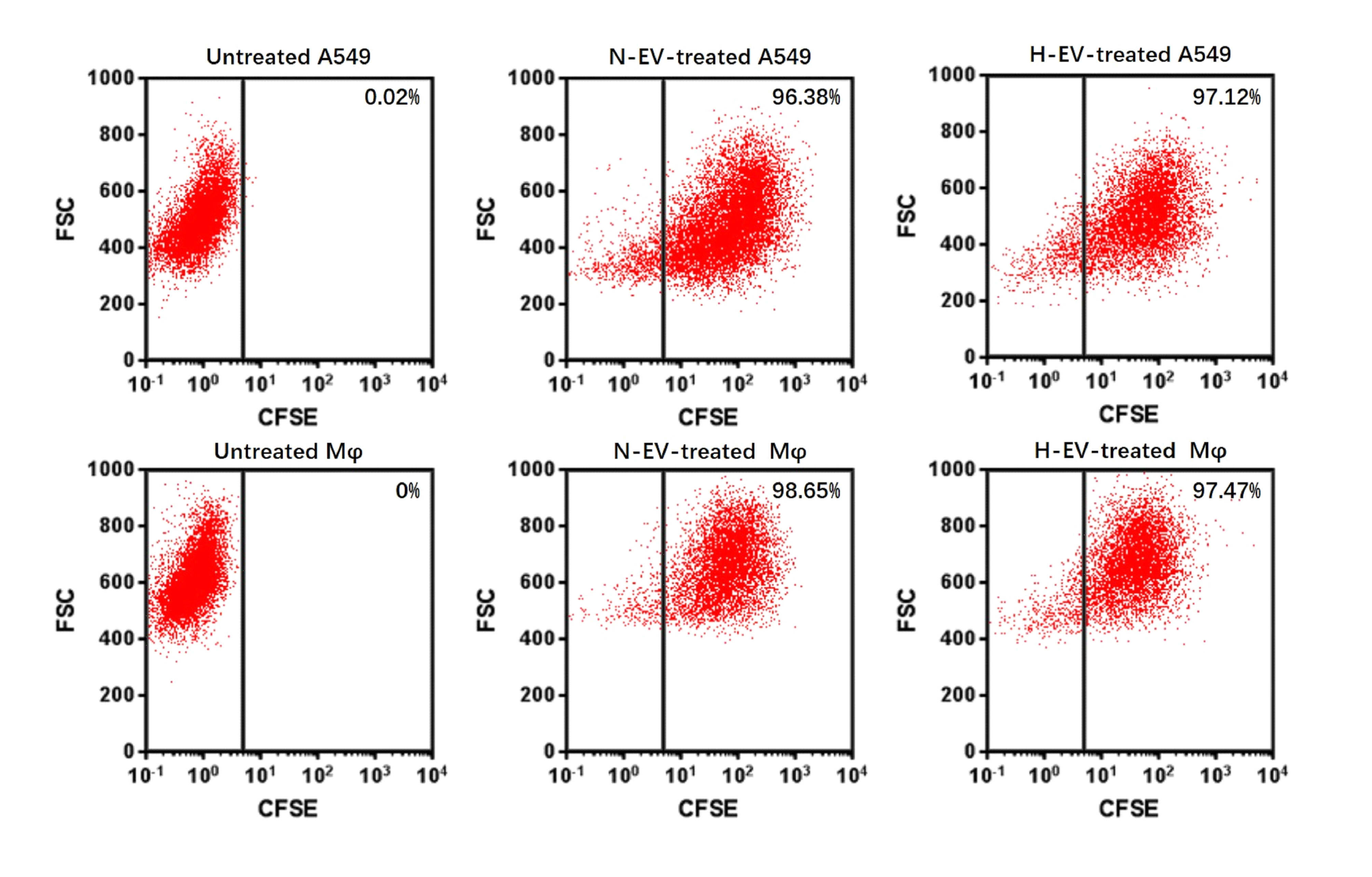

Supplement: Supplementary file 2 — Figure S2. Uptake of MSC-EV by A549 or human circulating monocytes as examples of recipients was confirmed by flow cytometry. Cells were treated with N-EV or H-EV that were labeled with CFSE dye before treatment, and cellular fluorescence was detected by flow cytometry. Cells processed in parallel using vehicle (sterile PBS) was used as negative control. (TIF 4714 kb) [file 13046_2019_1027_MOESM2_ESM.tif]
